# Postmortem Antigen-Detecting Rapid Diagnostic Tests to Predict Infectivity of SARS-CoV-2–Associated Deaths

**DOI:** 10.3201/eid2801.211749

**Published:** 2022-01

**Authors:** Fabian Heinrich, Ann Sophie Schröder, Anna-Lina Gerberding, Moritz Gerling, Felicia Langenwalder, Philine Lange, Axel Heinemann, Eric Bibiza-Freiwald, Dominik Sebastian Nörz, Martin Aepfelbacher, Susanne Pfefferle, Benjamin Ondruschka, Marc Lütgehetmann

**Affiliations:** University Medical Center Hamburg-Eppendorf, Hamburg, Germany

**Keywords:** COVID-19, SARS-CoV-2, antigen-detecting rapid diagnostic test, Ag-RDT, coronavirus disease 2019, infectivity, postmortem, respiratory infections, serostatus, severe acute respiratory syndrome coronavirus 2, viral RNA load, viruses, zoonoses, coronavirus

## Abstract

We investigated the infectivity of 128 severe acute respiratory disease coronavirus 2–associated deaths and evaluated predictive values of standard diagnostic procedures. Maintained infectivity (20%) did not correlate with viral RNA loads but correlated well with anti-S antibody levels. Sensitivity >90% for antigen-detecting rapid diagnostic tests supports their usefulness for assessment.

Deaths associated with severe acute respiratory syndrome coronavirus 2 (SARS-CoV-2) have raised concerns that contact with the corpses of deceased persons might pose a risk for transmitting infection (*1*). Nasopharyngeal SARS-CoV-2 RNA loads were shown to remain stable up to 20 days postmortem (*2*), and the maintained infectivity of corpses has sporadically been examined (*2*–*4*). In contrast, body surfaces of corpses have been considered noninfectious (*5*). Systematic studies on the infectivity of corpses and predictive values of standard diagnostic procedures remain scarce.

For this study, we prospectively collected nasopharyngeal swab specimens from 128 SARS-CoV-2 RNA-positive and 72 RNA-negative corpses <14 days postmortem to assess infectivity and predictive values of virologic parameters (Table). We excluded corpses exhibiting advanced putrefaction. For initial assessment, we determined RNA loads using quantitative reverse transcription PCR (qRT-PCR) (Appendix).

**Table:** Baseline characteristics of corpses received by the Institute of Legal Medicine, Hamburg, Germany, 2020–2021*

**Characteristic**	**SARS-CoV-2 RNA positive,† n = 128**	**SARS-CoV-2 RNA negative,† n = 72**	**Total, n = 200**
Age, y, median (IQR)	83.5 (71.5–89.1)	81.0 (73.0–87.0)	82.3 (72.9–88.5)
Sex			
M	71 (55.5)	36 (50.0)	107 (53.5)
F	57 (44.5)	36 (50.0)	93 (46.5)
Place of death			
Home	28 (22.0)	30 (41.7)	58 (29.1)
Nursing home	38 (29.9)	3 (4.2)	41 (20.6)
Hospital	39 (30.7)	25 (34.7)	64 (32.2)
ICU	20 (15.7)	10 (13.9)	30 (15.1)
Other	2 (1.6)	4 (5.6)	6 (3.0)
Postmortem interval,‡ h, median (IQR)	8.7 (5.3–82.6)	4.9 (3.5–8.8)	7.0 (4.3–49.9)
Putrefactive changes	11 (8.9)	1 (1.4)	12 (6.1)
SARS-CoV-2 RNA load,¶ copies/mL, median (IQR)	7.0 x 10^6^ (5.5 × 10^4^–5.2 x 10^7^)	Below LOD	Not applicable

*Values are no. (%) except as indicated. In case of missing data points, valid percentages are indicated. ICU, Intensive care unit; LOD, limit of detection; SARS-CoV-2, severe acute respiratory syndrome coronavirus 2 †B.1.1.7 variants (2/128) identified by multiplex-typing PCR (*5*). SARS-CoV-2–associated deaths were tested in a multiplex typing PCR for SARS-CoV-2 spike variants. ‡Interval from time of death until initial sampling and cooling at 4°C.

We found SARS-CoV-2 RNA up to 325 hours postmortem, but RNA loads did not correlate with the postmortem interval (PMI; r = 0.003, p >0.99) (Figure, panel A). RNA loads were comparatively high (median 7.0 × 10^6^ copies/mL, interquartile range [IQR] 5.5 × 10^4^–5.2 × 10^7^ copies/mL) (Figure, panel B) and in some cases exceeded loads in the acute phase of the disease (*6*), possibly because of postmortem mucosal softening and higher exfoliation of tissue during sample collection.

**Figure:**
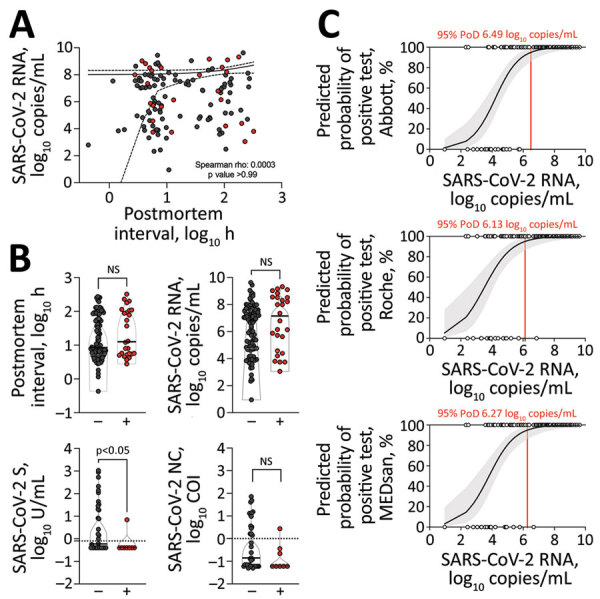
Overview of 128 consecutive records of SARS-CoV-2–associated deaths received by the Institute of Legal Medicine, Hamburg, Germany, 2020–2021. A) SARS-CoV-2 RNA loads by postmortem intervals. Spearman correlation was performed; estimates and 95% CI are shown. B) Postmortem intervals, viral RNA loads, quantitative (S), and qualitative (NC) antibody levels compared among culture-positive (+) and culture-negative (–) corpses. Comparisons were performed using Mann-Whitney-U or χ^2^ testing, as appropriate. Median and interquartile ranges are shown. Horizontal dotted lines indicate cutoff value. C) Probability of positive antigen-detecting rapid diagnostic test results depending on viral RNA loads calculated by binomial logistic regression. Robust estimates with 95% CI are shown. Vertical red line indicates 95% PoD with the corresponding viral RNA load. Ag-RDT, antigen-detecting rapid antigen test; COI, cut-off index; NC, nucleocapsid; NS, not significant; PoD, probability of detection; S, spike; SARS-CoV-2, severe acute respiratory syndrome coronavirus 2.

Virus isolation proved infectivity was maintained in 26/128 (20%) corpses (Appendix). PMI (median 13 hours, range 3–325 hours) and SARS-CoV-2 RNA load (1.4 × 10^7^ copies/mL, IQR 3.7 × 10^4^–3.3 × 10^8^) among culture-positive corpses did not differ significantly from PMI (median 8 hour, range 0–275 hour; p = 0.38) and RNA loads (7.0 × 10^6^ copies/mL, IQR 5.8 × 10^4^–3.9 × 10^7^ copies/mL; p = 0.14) among culture-negative corpses (Figure, panel B). We successfully isolated virus from samples with comparatively low amounts of RNA (<1 × 10^4^ copies/mL), in contrast with previous findings among living patients (*6*). We observed putrefactive changes in no culture-positive corpses compared with in 11/98 (11%) culture-negative corpses (χ^2^ = 3.20; p = 0.11), indicative of potentially decreased infectivity.

We confirmed seroconversion in 18/44 (41%) blood samples, 15/43 (35%) anti-nucleocapsid positive and 17/44 (39%) anti-spike positive (range <0.4–1066.0 U/mL; Appendix). Levels of anti-spike antibodies, representing neutralizing antibody levels (*7*), were not significantly correlated with PMI (r = 0.07; p = 0.64), but were well correlated with viral RNA levels (r = –0.70; p <0.0001). Anti-nucleocapsid antibodies were found in only 1/8 (13%) culture-positive compared with 14/35 (40%) culture-negative corpses (χ^2^ = 2.17; p = 0.23) (Figure, panel C). Moreover, anti-spike antibody levels differed significantly (p = 0.04) between culture-positive (1.22 U/mL, SD 2.32) and culture-negative (86.85 U/mL, SD 240.56) corpses, indicative of inverse association of SARS-CoV-2–specific antibody levels with infectivity (Figure, panel C).

Antigen-detecting rapid diagnostic tests (Ag-RDTs) are considered adequate alternative swift diagnostic tools in living patients (*8*,*9*), but knowledge about their postmortem applicability and reliability remains scarce. We tested Ag-RDTs from 3 manufacturers and found excellent performance for postmortem use (Appendix Table 1). Compared with qRT-PCR results, for the Panbio COVID-19 Ag Rapid Test Device (Abbott, https://www.abbott.com), sensitivity was 80.3% (95% CI 72.3%–86.4%) and specificity 100.0% (95% CI 95.0%–100.0%); for the SARS-CoV-2 Rapid Antigen Test (Roche https://www.roche.com), sensitivity was 86.4% (95% CI 79.1%–91.9%) and specificity 98.6% (95% CI 93.0%–100.0%); and for the SARS-CoV-2 Antigen Rapid Test (MEDsan https://www.medsan.eu), sensitivity was 84.1% (95% CI 76.6%–90.0%) and specificity 95.8% (95% CI 88.0%–99.0%) (Appendix Figures 1, 2).

We found SARS-CoV-2 RNA load correlated with Ag-RDT positivity in univariate and multivariate analyses (p<0.001), thereby confirming their predictive value (Figure, panel C; Appendix Table 2). Subgroup analyses of corpses with >1 × 10^6^ RNA copies/mL (n = 74) revealed 100% (95% CI 95.1%–100.0%) sensitivity in Abbott (n = 74) and Roche and MEDsan (n = 73 each) assays. In contrast, neither PMI (p = 0.34) nor putrefactive changes (p = 0.90) were predictive for testing positive in Ag-RDTs (exemplarily for the MEDsan assay; Appendix Table 2). Ag-RDT sensitivity in infectious corpses was 92.3% (95% CI 74.9%–99.1%) for Abbott, 96.2% (95% CI 80.4%–99.9%) for Roche, and 96.2% (95% CI 80.4%–99.9%) for MEDsan. We detected 2 SARS-CoV-2 variants of concern despite relatively low viral RNA loads (4.83 log_10_); the 2 samples tested positive by Abbott and Roche but were missed by MEDsan.

The first limitation of our study is that blood was not available from all corpses, and the serologic assays and Ag-RDTs used are not approved for cadaveric samples. Furthermore, because of a shortage of reagents and supplies, we had to use different tests to quantify RNA, and slight deviations cannot be ruled out.

In summary, we show that cadavers from SARS-CoV-2–associated deaths remain infectious long after death in a considerable proportion of cases. Postmortem infectivity does not correlate with PMI or viral RNA load but correlates with the absence of virus-specific antibodies. Ag-RDTs performed well, enabling rapid on-site detection. Because previous studies among living patients indicate that Ag-RDTs reliably detect all SARS-CoV-2 variants (*10*), we believe that our results on postmortem Ag-RDTs use can contribute to crisis management in severely affected regions and increase safety in the medical sector worldwide.

Appendix Additional information about study of infectivity of cadavers from SARS-COV-2 deaths
