# Supplementary material for: Postmortem Antigen-Detecting Rapid Diagnostic Tests to Predict Infectivity of SARS-CoV-2–Associated Deaths
Source: Emerg Infect Dis. 2022 Jan;28(1):244–7. doi: 10.3201/eid2801.211749 (PMC8714232; doi:10.3201/eid2801.211749)
Supplement: Appendix — Additional information about study of infectivity of cadavers from SARS-COV-2 deaths [file 21-1749-Techapp-s1.pdf]

Article DOI: https://doi.org/10.3201/eid2801.211749

Postmortem Antigen-Detecting Rapid Diagnostic Tests to Predict Infectivity of SARS-CoV-2–Associated Deaths

Appendix

Methods

Patients and Ethics

For the study, we prospectively included a total of 200 corpses received at the Institute of Legal Medicine (University Medical Center Hamburg-Eppendorf, Hamburg, Germany); we excluded corpses exhibiting advanced putrefactive changes (marbling and mummification). All corpses were stored at 4°C upon receipt; we defined postmortem interval as the time from death until cooling. Informed consent was obtained from relatives or legal representatives. We performed data and sample acquisition from November 1, 2020–February 28, 2021. For initial assessment for SARS-CoV-2 RNA, quantitative reverse transcription (qRT-) PCR from nasopharyngeal swab samples was performed as part of routine diagnostics at the Institute of Microbiology, Virology and Hygiene (University Medical Center Hamburg-Eppendorf, Hamburg, Germany). In total, 128/200 corpses were SARS-CoV-2 RNA positive, and 72/200 were SARS-CoV-2 RNA negative. Notably, none of the 72 SARS-CoV-2 RNA–negative deceased patients had had a diagnosis of COVID-19 during their lifetime nor did they have a diagnosed or suspected case of SARS-CoV-2 or COVID-19 at the time of death.

Sampling and Molecular Diagnostic

We performed an initial assessment for the presence of SARS-CoV-2 RNA in all corpses received at the Institute of Legal Medicine by qRT-PCR. Following receipt of the initial results (usually <24 h later), we performed, 4 subsequent nasopharyngeal swabs, 1 tested using universal transport medium (MANTACC, https://www.mantacc.com) for qRT-PCR and virus isolation, and 3 for antigen-detecting rapid diagnostic tests using the swab supplied with the kit. For quantitative SARS-CoV-2 RNA detection, we used commercially available assays, such as

Cepheid Xpert Xpress SARS-CoV-2 (https://www.cepheid.com), Roche cobas SARS-CoV-2 (https://www.roche.com), and lab-developed assays (*1*,*2*). We used standard RNA reference material (obtained from INSTAND eV, https://www.instand-ev.de) for quantification. To calculate $\log_{10}$ RNA copies/mL (y) based on Ct-values (x), targets and conversion formulae were used: Cepheid Xpert Xpress SARS-CoV-2: $y = -0.29x+12.83$ (target E2); Roche cobas SARS-CoV-2: $y = -0.308x+13.81$ (target T2); SARS-CoV-2_UCT (utility channel test) LDT (lab-developed test): $y = -0.291x+12.97$ (target E-gene); NeuMoDx LDT: $y = -0.425x+14.8$ (https://www.neumodx.com; target E-gene), Roche LightCycler 480 II: $y = -0.318x+13.32$ (target E-gene). We did not consider the nonlinearity of RNA quantification within the analysis. We also analyzed all nasopharyngeal swab samples in a multiplex typing PCR (*3*), detecting del 69/70 and 501Y, enabling us to distinguish SARS-CoV-2 spike variants of concern, such as B.1.1.7 and B.1.351.

Cell Culture and Virus Isolation

We maintained and cultivated Vero E6 cells under standard conditions (*4*). For virus isolation, we used 500µL of each swab medium (universal transport medium) taken at the time of antigen-detecting rapid diagnostic (Ag-RDT) testing, and performed infection as described elsewhere (*5*). We analyzed virus growth after incubation at 37°C for 72h by qRT-PCR as described elsewhere (*1*).

Serologic Diagnostic

We obtained cadaveric blood from all corpses evaluated by full autopsy, 44/128 SARS-CoV-2 RNA–positive corpses. We used Roche Elecsys Anti-SARS-CoV-2-NC with the Roche cobas e411 according to manufacturer recommendations, for qualitative detection of SARS-CoV-2 nucleocapsid protein antibodies. We used Roche Elecsys Anti-SARS-CoV-2-S with the Roche cobas e411 according to manufacturer recommendations, for the quantitative detection of SARS-CoV-2 spike antibodies. We set cutoff values according to manufacturer recommendations: >1 COI (Elecsys Anti-SARS-CoV-2-NC) and >0.8 U/mL (Elecsys Anti-SARS-CoV-2-S).

Evaluation of Ag-RDTs

We performed Ag-RDTs from 3 different manufacturers (Appendix Table 1) according to manufacturer protocols: I) Abbott Panbio COVID-19 Ag Rapid Test Device

(https://www.abbott.com), II) Roche SARS-CoV-2 Rapid Antigen Test (https://www.roche.com), and III) MEDsan SARS-CoV-2 Antigen Rapid Test (https://www.medsan.eu). All 3 Ag-RDTs detect the SARS-CoV-2 nucleoprotein (N). All assays were listed by official authorities to meet the requirements for SARS-CoV-2 testing in Germany (*6*), but none of them was approved for use in the postmortem setting. Two independent examiners performed Ag-RDT readouts by visual inspection.

Statistical Analysis

We performed a sample size estimation for the number of cases included, assuming a significance level of $\alpha = 0.05$ and applying a margin of error of 0.05. We tested data distribution and variance equality by Q-Q plot and homoscedasticity plot. We used a Mann-Whitney-U test to compare differences between 2 independent groups in nonparametric distributed, unpaired datasets. We used χ^2 testing to compare proportions between groups. We calculated Spearman's rank correlation coefficients to assess the statistical correlation of nonparametric distributed variables. We used binary logistic regression and multivariate logistic regression for multivariate analyses. We included independent variables in the model on a clinical and scientific basis. We calculated Clopper-Pearson 95% confidence intervals for binomial proportions. P values <0.05 were considered statistically significant. We performed statistical analysis using IBM SPSS Statistics, version 27.0.0.0 (https://www.ibm.com), and STATA/MP, version 17.0 (https://www.stata.com). We used GraphPad Prism software version 9.1.1 (https://www.graphpad.com) for data illustration.

References

1. Pfefferle S, Reucher S, Nörz D, Lütgehetmann M. Evaluation of a quantitative RT-PCR assay for the detection of the emerging coronavirus SARS-CoV-2 using a high throughput system. [Erratum in Euro Surveill. 2020;25:20200312c.]. Euro Surveill. 2020;25:2000152. PubMed https://doi.org/10.2807/1560-7917.ES.2020.25.9.2000152

2. Nörz D, Fischer N, Schultze A, Kluge S, Mayer-Runge U, Aepfelbacher M, et al. Clinical evaluation of a SARS-CoV-2 RT-PCR assay on a fully automated system for rapid on-demand testing in the hospital setting. J Clin Virol. 2020;128:104390. PubMed https://doi.org/10.1016/j.jcv.2020.104390

3. Nörz D, Grunwald M, Olearo F, Fischer N, Aepfelbacher M, Pfefferle S, et al. Evaluation of a fully automated high-throughput SARS-CoV-2 multiplex qPCR assay with built-in screening functionality for del-HV69/70- and N501Y variants such as B.1.1.7. J Clin Virol. 2021;141:104894. PubMed https://doi.org/10.1016/j.jcv.2021.104894

4. Ammerman NC, Beier-Sexton M, Azad AF. Growth and maintenance of Vero cell lines. Curr Protoc Microbiol. 2008;Appendix 4:Appendix 4E.

5. Brehm TT, Pfefferle S, von Possel R, Kobbe R, Nörz D, Schmiedel S, et al. SARS-CoV-2 reinfection in a healthcare worker despite the presence of detectable neutralizing antibodies. Viruses. 2021;13:661. PubMed https://doi.org/10.3390/v13040661

6. Federal Institute for Drugs and Medical Devices (BfArM). Antigen tests for SARS-CoV-2 [German]. [cited 2020 Aug 11] https://www.bfarm.de/EN/Medical-devices/Tasks/Special-topics/Antigen-tests/_node.html

Appendix Table 1. Antigen-detecting rapid diagnostic test specifications as provided by the manufacturer for all tests used in the study*

Test device name	Manufacturer	Sensitivity, % (95% CI)	Specificity, % (95% CI)	Limit of detection, $TCID_{50}$/ mL
Panbio COVID-19 Ag Rapid Test Device	Abbott†	93.3 (83.8–98.2)	99.4 (97.0–100.0)	1.50×10^2
SARS-CoV-2 Rapid Antigen Test	Roche Diagnostics Deutschland GmbH‡	96.5 (91.3–99.0)	99.7 (98.2–99.9)	4.94×10^2
MEDsan SARS-CoV-2 Antigen Rapid Test	MEDsan¶	92.5 (86.2–96.5)	99.8 (98.9–99.9)	1.40×10^1

*$TCID_{50}$, 50% tissue culture infection dose.
†https://www.abbott.com
‡https://www.roche.com
¶https://www.medsan.eu
.

Appendix Table 2. Predictive factors for positive testing by antigen-detecting rapid diagnostic tests investigated in univariate and multivariate logistic regression analyses*,†

Parameter	Univariate analysis OR (95%CI)	Univariate analysis P value#	Multivariate analysis OR (95%CI)	Multivariate analysis P value#
Abbott assay‡				
Postmortem interval, /h	1.00 (0.99–1.00)	0.70	1.00 (0.99–1.01)	0.70
SARS-CoV-2 RNA load, log_{10}, copies/mL	3.65 (2.16–6.17)	<0.0001	3.65 (2.14–6.23)	<0.0001
Putrefactive changes	1.55 (1.03–2.33)	0.04	1.34 (0.78–2.31)	0.29
Roche assay¶				
Postmortem interval, /h	1.01 (1.00–1.02)	0.15	1.01 (1.00–1.03)	0.09
SARS-CoV-2 RNA load, log_{10}, copies/mL	3.09 (1.81–5.28)	<0.0001	3.49 (1.95–6.25)	<0.0001
Putrefactive changes	1.22 (0.71–1.79)	0.63	0.66 (0.33–1.31)	0.23
MEDsan assay§				
Postmortem interval, /h	1.00 (0.99–1.01)	0.49	1.00 (1.00–1.01)	0.34
SARS-CoV-2 RNA load, log_{10}, copies/mL	3.31 (1.94–5.64)	<0.0001	3.40 (1.97–5.86)	<0.0001
Putrefactive changes	1.32 (0.89–1.95)	0.17	0.96 (0.56–1.65)	0.90

*OR, odds ratio; SARS-CoV-2, severe acute respiratory syndrome coronavirus 2
†True-positive testing served as the dependent variable (compared with qRT-PCR). Independent variables in the model were included on a clinical and scientific basis.
‡https://www.abbott.com; model estimator: $\chi^2 = 56.11$, $p < 0.0001$.
¶https://www.roche.com; model estimator: $\chi^2 = 41.86$, $p < 0.0001$.
§https://www.medsan.eu; model estimator: $\chi^2 = 44.22$, $p < 0.0001$.
#P values <0.05 considered statistically significant.

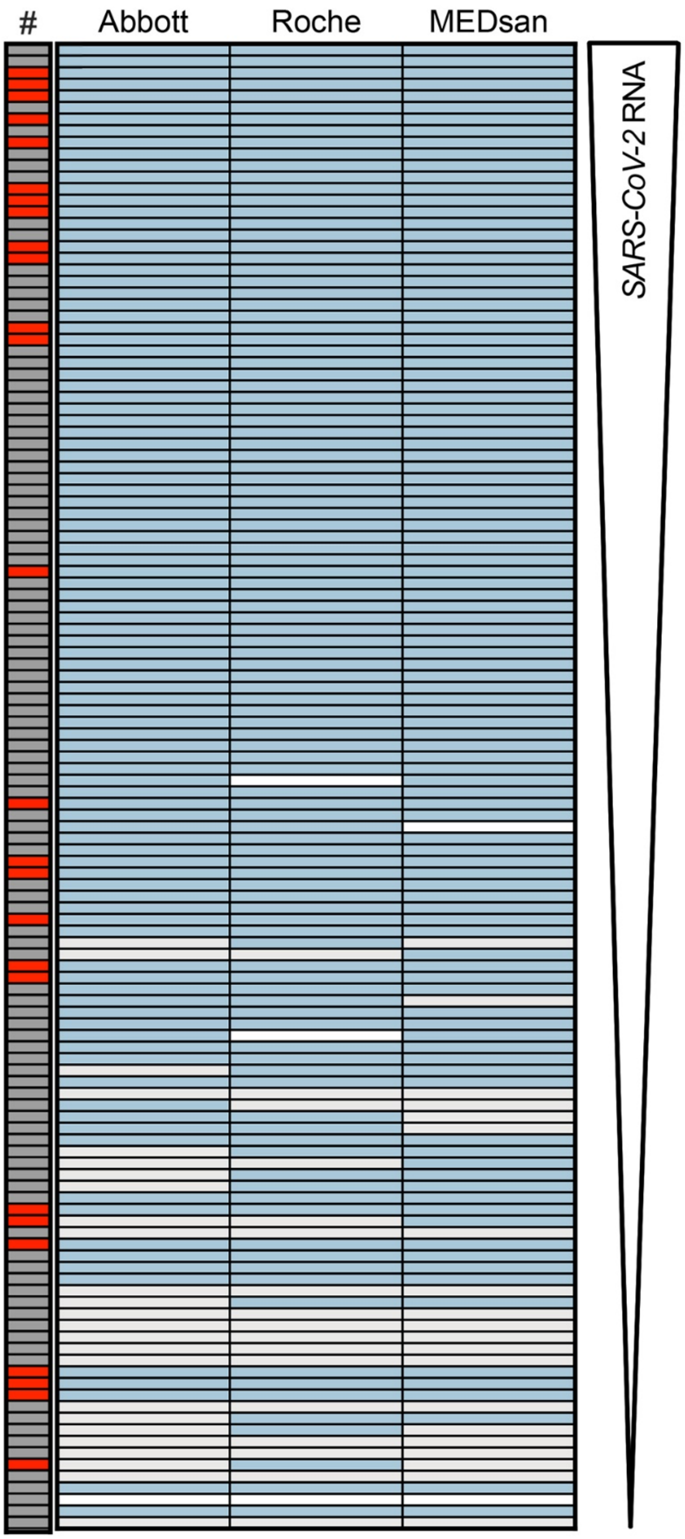

Appendix Figure 1. Antigen-detecting rapid diagnostic test results are illustrated as positive (light blue) and negative (light gray). # indicates virus culture status; culture-positive corpses are marked red.

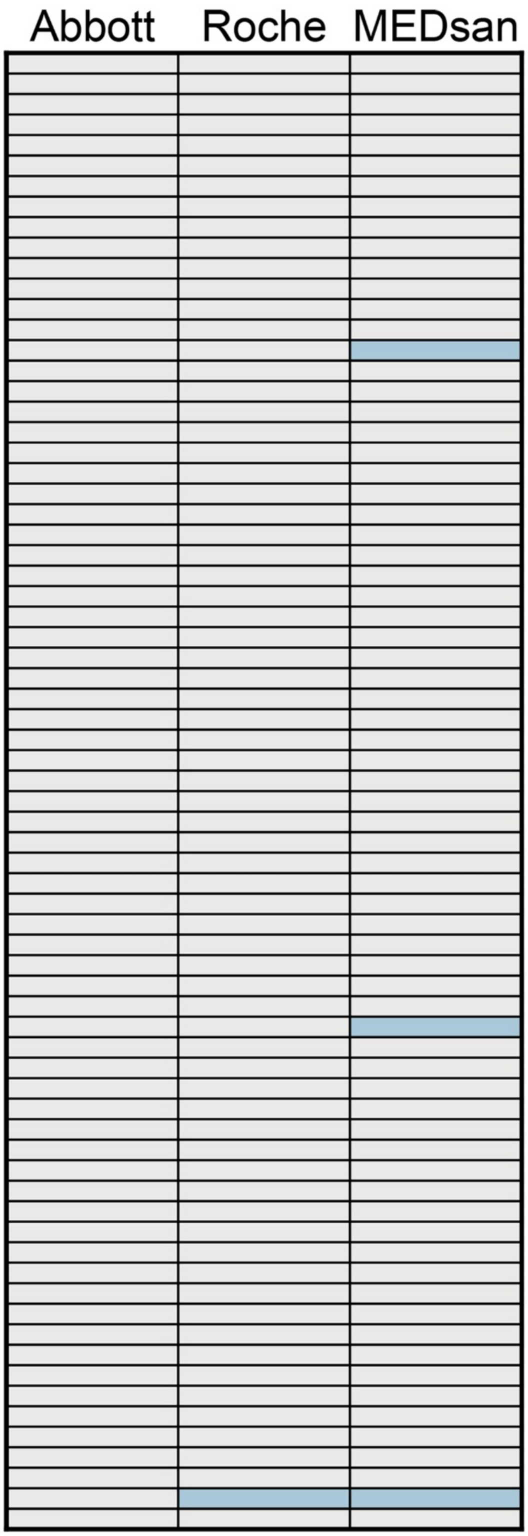

Appendix Figure 2. Specificity of antigen-detecting rapid diagnostic tests in the postmortem setting. Overview of test results in SARS-CoV-2–negative corpses (n = 72). Positive test results are marked in light blue and negative results in light gray.
